# Increased Risk for Group B *Streptococcus* Sepsis in Young Infants Exposed to HIV, Soweto, South Africa, 2004–2008^1^

**DOI:** 10.3201/eid2104.141562

**Published:** 2015-04

**Authors:** Clare L. Cutland, Stephanie J. Schrag, Michael C. Thigpen, Sithembiso C. Velaphi, Jeannette Wadula, Peter V. Adrian, Locadiah Kuwanda, Michelle J. Groome, Eckhart Buchmann, Shabir A. Madhi

**Affiliations:** University of the Witwatersrand, Johannesburg, South Africa (C.L. Cutland, S.C. Velaphi, J. Wadula, P.V. Adrian, L. Kuwanda, M.J. Groome, E. Buchmann, S.A. Madhi);; Medical Research Council: Respiratory and Meningeal Pathogens Research Unit, Johannesburg (C.L. Cutland, P.V. Adrian, L. Kuwanda, M.J. Groome, S.A. Madhi);; Centers for Disease Control and Prevention, Atlanta, Georgia, USA (S.J. Schrag, M.C. Thigpen);; Chris Hani Baragwanath Academic Hospital, Johannesburg (S.C. Velaphi, J. Wadula, E. Buchmann);; National Institute for Communicable Diseases, Sandringham, South Africa (S.A. Madhi)

**Keywords:** group B Streptococcus, GBS, early-onset disease, late-onset disease, HIV, serotype, bacteria, viruses, South Africa, sepsis, exposure, increased risk, vaccine, infants, pregnant women, epidemiology, invasive disease, streptococci

## Abstract

Although group B *Streptococcus* (GBS) is a leading cause of severe invasive disease in young infants worldwide, epidemiologic data and knowledge about risk factors for the disease are lacking from low- to middle-income countries. To determine the epidemiology of invasive GBS disease among young infants in a setting with high maternal HIV infection, we conducted hospital-based surveillance during 2004–2008 in Soweto, South Africa. Overall GBS incidence was 2.72 cases/1,000 live births (1.50 and 1.22, respectively, among infants with early-onset disease [EOD] and late-onset [LOD] disease). Risk for EOD and LOD was higher for HIV-exposed than HIV-unexposed infants. GBS serotypes Ia and III accounted for 84.0% of cases, and 16.9% of infected infants died. We estimate that use of trivalent GBS vaccine (serotypes Ia, Ib, and III) could prevent 2,105 invasive GBS cases and 278 deaths annually among infants in South Africa; therefore, vaccination of all pregnant women in this country should be explored.

## Abstract 2

Vaccination of pregnant women could prevent 2,105 invasive GBS cases and 278 deaths among infants annually.

In 2013, a total of 41.6% (2.6 million) of deaths worldwide in children <5 years of age occurred in neonates; 76.7% occurred within 6 days after birth (*1*). Furthermore, in 2012, ≈6.9 million probable cases of severe bacterial infections and 680,000 associated deaths occurred among neonates (*2*). Nevertheless, there is a paucity of data from low- and middle-income countries on pathogen-specific causes of neonatal sepsis, particularly during the first day of life, and it is unknown whether in utero HIV exposure increases susceptibility to severe neonatal bacterial infections.

Group B *Streptococcus* (GBS) is a leading cause of severe invasive disease in young infants. A meta-analysis dominated by studies from high-income countries estimated global incidence of 0.53 cases/1,000 live births during 2000–2011 (*3*). Considerable intra- and interregional variation in the incidence of invasive early-onset disease (EOD; disease in 0- to 6-day-old infants ) was observed (*3*–*5*), ranging from 1.21 cases/1,000 live births (95% CI 0.5–1.91) in Africa to 0.02 cases/1,000 live births (95% CI 0–0.07) in Southeast Asia (*3*). This variability is inconsistent with the lesser difference in prevalence of maternal GBS colonization, the major risk factor for EOD, in women from different regions (20.9% in Africa, 13.4% in Southeast Asia) (*6*). Maternal HIV infection has not been associated with a higher prevalence of GBS colonization (*7*,*8*), except among women with CD4+ lymphocyte counts of >500 cells/mm^3^ (*8*).

Providing intrapartum antimicrobial drug prophylaxis (IAP) to women identified as rectovaginally colonized by GBS at 35–37 weeks’ of pregnancy has been associated with a >80.0% reduction in EOD (*9*); however, this strategy is logistically challenging to implement and maintain in resource-constrained settings. Furthermore, IAP has not decreased the incidence of late-onset disease (LOD; disease in 7- to 90-day-old infants) (*10*).

Progress has been made in the development of a trivalent GBS polysaccharide–protein conjugate vaccine (GBS-CV), which is targeted for use in pregnant women; the goal is to enhance transplacental transfer of capsular antibody to the fetus (*1*,*8*–*10*), which could protect against EOD and LOD. Improved estimates of the incidence of invasive GBS disease are needed from low- and middle-income countries to contextualize the prioritization of GBS vaccination and determine whether temporal changes in invasive serotypes should be considered in the design of serotype-specific GBS vaccine.

We evaluated the clinical and microbiological epidemiology, incidence, and serotype distribution of invasive GBS disease among young infants in a setting with a high prevalence of maternal HIV infection. A secondary aim was to estimate the potential effect of a trivalent GBS-CV in reducing the number of invasive GBS cases nationally.

## Materials and Methods

### Study Setting and Design

During 2004–2008, we undertook hospital-based surveillance of culture-confirmed invasive bacterial sepsis in infants 0–90 days of age at Chris Hani Baragwanath Academic Hospital (CHBAH), a public secondary–tertiary health care facility in Soweto, South Africa. CHBAH is the only public hospital in Soweto with neonatal care facilities; ≈90.0% of all hospitalizations from the community occur in this hospital. Soweto is a predominantly black-African community and has 1.4 million inhabitants, including 125,000 children <5 years of age and a birth cohort of ≈28,000/year (*11*); ≈21,000 are delivered at CHBAH and 7,000 are delivered at 1 of 6 community-based midwife obstetric units. Women with potentially complicated deliveries at midwife obstetric units and clinically ill newborns are referred to CHBAH by ambulance. 

Health care for pregnant women and children is provided free of charge in South Africa (*12*). Most deliveries in Soweto (95.0%) (E. Buchmann, pers. comm., 2014 Jul 19) and in South Africa as a whole (87.3% in 2010) (*13*) occur in health facilities. Voluntary counseling and testing for HIV is offered at antenatal clinics; >96.0% of pregnant women accept testing (C. Mnyani, pers. comm,, 2014 Jul 28). Single-dose nevirapine, administered as standard of care to women in labor and their newborns to prevent mother-to-child HIV transmission, was supplemented in 2007 with triple antiretroviral therapy to immunocompromised women (<350 CD4+ cells/mm^3^) from 34 weeks’ gestation onward.

During the surveillance period, HIV prevalence in pregnant women remained stable at 29.9% (*14*), and ≈18.0% of all children were born prematurely (<37 weeks’ gestational age) or had a low birthweight (<2,500 g) (CHBAH, unpub. data). Gestational age was determined on the basis of the available obstetric or neonatal assessments by attending physicians. Healthy newborns are routinely discharged home 12 h after vaginal or 72 h after cesarian delivery.

At CHBAH, newborns with signs and symptoms of severe illness at birth or before discharge from postnatal wards are admitted to the neonatal unit; infants who are discharged home after delivery and subsequently return for suspected bacterial infections are hospitalized in the general pediatric wards. Investigation and treatment of neonates and young infants with suspected invasive bacterial disease were conducted according to standard of care by attending physicians. Investigations included complete blood cell counts and blood cultures for all infants. Lumbar punctures (to obtain cerebrospinal fluid [CSF] samples for biochemistry, microscopy, and antimicrobial drug sensitivity testing and culture) were limited to infants with GBS-positive cultures of blood samples obtained at birth and to all infants admitted from the community for suspected sepsis. Sterile-site cultures were processed at the National Health Laboratory Service (NHLS). Blood cultures were evaluated by using the BacT/Alert microbial system (Organon Teknika, Durham, NC, USA). GBS isolates were retrieved from NHLS, stored at −70°C, and serotyped by latex agglutination (*15*). During the surveillance period, empiric treatment for suspected sepsis consisted of intravenous penicillin and gentamicin for neonates and ampicillin and gentamicin for infants 1–12 months of age; case-patients with suspected meningitis received ampicillin and cefotaxime empirically.

Maternal screening for rectovaginal GBS colonization during pregnancy is not routinely performed in public health facilities in South Africa. Before 2007, CHBAH IAP guidelines recommended administration of intravenous ampicillin (1 g/6 h) and oral metronidazole (400 mg 3×/d) for suspected chorioamnionitis and prolonged rupture of membranes. In January 2007, targeted risk-based IAP was implemented for possible GBS infection in women with preterm labor, a previous infant infected with GBS, or a GBS-positive culture; treatment consisted of an initial 2-g dose of intravenous ampicillin, followed by intravenous ampicillin (1 g/4 h) until delivery. During the surveillance period, 10.5% of women received IAP during labor (*16*).

Infants 0–90 days of age who were admitted to CHBAH with GBS isolated from a normally sterile site were identified through screening of ward admissions and microbiological records within 24 h of identification of GBS. We also undertook an audit of the NHLS database to identify all invasive pathogens isolated from infants over the study period. Invasive GBS disease was categorized as bacteremia if identified in blood only and as meningitis if identified from CSF or if there was CSF cytologic evidence of purulent meningitis (>5 leukocytes/mm^3^; adjusted in traumatic lumbar punctures to allow 1 leukocyte/500 erythrocytes) in an infant with GBS bacteremia.

Demographic, birth, maternal HIV infection status, and other clinical data were abstracted from infants’ medical records by study doctors. HIV exposure was determined by abstracting antenatal HIV test results of mothers and supplemented by HIV ELISA results from maternal or infant blood tests conducted by attending physicians. The HIV infection status of HIV-exposed infants was determined by using a qualitative HIV PCR at the discretion of the attending physician.

### Statistical Considerations

Incidence was calculated as cases per 1,000 live births. Administrative live birth data from CHBAH and community clinics for Soweto and HIV prevalence survey data for the surveillance period were used to determine population denominators for HIV-infected and -uninfected women. Established patient referral protocols limited the chance that neonates, especially newborns, living outside the hospital catchment area were admitted to CHBAH. A sensitivity analysis of incidence was undertaken to account for GBS case-patients for whom maternal HIV status was unknown. For overall and annual incidence calculations, we attributed the prevalence of HIV exposure among case-patients with known exposure status to case-patients with unknown exposure status. Alternate sensitivity analyses assumed all cases with unknown maternal HIV status were HIV-exposed or, conversely, HIV-unexposed.

On the basis of 2012 population data for number of live births in South Africa (1,168,403) (*17*) and national prevalence of HIV infection in pregnant women (29.5%) (*18*), we used our study data to estimate annual national number of invasive GBS cases and deaths and stratified estimates by HIV exposure. We estimated the number of annual vaccine-preventable invasive GBS cases and deaths in South Africa on the basis of the conservative assumption that administration of GBS-CV to pregnant women (*19*) would not protect against invasive GBS disease in infants born at <33 weeks’ gestation, and we adjusted for the proportion of serotypes included in the current experimental trivalent GBS-CV (Ia, Ib, and III) and a future pentavalent vaccine (addition of serotypes II and V). These estimates were based on hypothetical vaccine efficacy assumptions of 75.0% for the overall population; 85.0% for HIV-unexposed infants; and 65.0% for HIV-exposed infants, as determined on the basis of the lower immunogenicity of trivalent GBS-CV in HIV-infected pregnant women (*19*).

Proportions were compared by using the χ^2^ and Fisher exact tests, as appropriate; Wilcoxon rank sum test (nonparametric) was applied for continuous variables. Univariate analysis was performed to determine factors associated with GBS-related death. Two-sided p values <0.05 were considered statistically significant, and 95% CIs were calculated. Analyses were conducted by using STATA/IC 13.0 (StataCorp, College Station, TX, USA).

### Ethics Consideration

The study was approved by the Human Research Ethics Committee, University of the Witwatersrand (M03–10–07 and M10–367) and the institutional review board of the Centers for Disease Control and Prevention. Mothers of infants prospectively identified with invasive GBS disease signed informed, written consent. Consent was waived by the ethics committees for retrospective review of records of cases identified after discharge or death of the infant.

## Results

**Figure 1:**
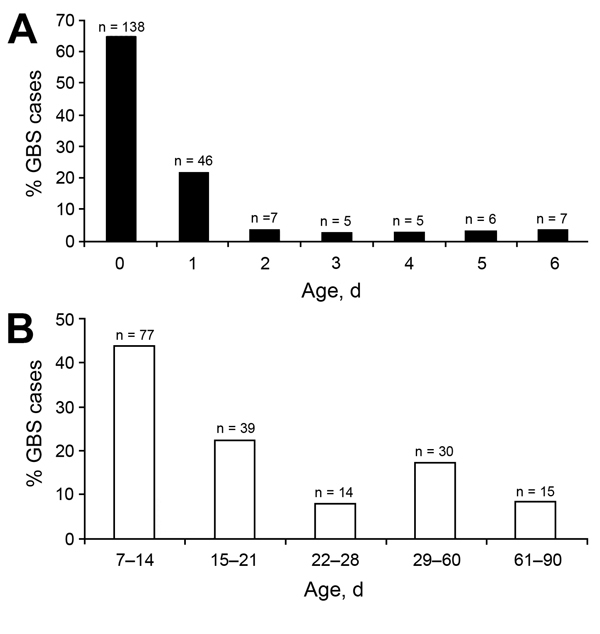
Age distribution of young infants (0–90 days of age) with invasive group B *Streptococcus* (GBS) sepsis, Soweto, South Africa, 2004–2008. A) Distribution for 214 infants with early-onset disease. B) Distribution for 175 infants with late-onset disease.

During the surveillance period, 389 invasive GBS cases were identified in infants 0–90 days of age; 214 (55.0%) cases were EOD. Complete medical records were unavailable for 17 cases (10 EOD, 7 LOD), which were included in incidence calculations but not in univariable analysis. Overall incidence of invasive GBS was 2.72 cases/1,000 live births (95% CI 2.46–3.01). EOD incidence was 1.50 cases/1,000 live births (95% CI 1.30–1.71), and LOD incidence was 1.22 cases/1,000 live births (95% CI 1.05–1.42); incidences for both were generally similar across years (data not shown). Overall, 26.6% of case-patients were born prematurely, including 29.8% of EOD and 22.3% of LOD case-patients. Most (69.4%) preterm births occurred at <33 weeks’ gestation, including 63.0% and 80.6% of those for EOD and LOD case-patients, respectively (Technical Appendix Table 1). Of the 214 EOD case-patients, 138 (64.5%) had positive culture results for blood samples obtained at birth (median age 0 days, interquartile range 0–1; Figure 1, panel A). Forty-four percent of LOD cases were detected during week 2 of life (median age 16 days, interquartile range 11–29; Figure 1, panel B). Infants with LOD were 5.57-fold (95% CI 3.50–8.90) more likely than infants with EOD to have meningitis (61.7% vs. 22.4%; p<0.0001).

**Table 1:** Incidence of invasive group B *Streptococcus* sepsis in 0- to 90-day-old infants, by in utero exposure to HIV, Soweto, South Africa, 2004–2008*

HIV exposure status	Overall			Bacteremia			Meningitis	
	No. cases, incidence (95% CI)†	RR (95 % CI)		No. cases, incidence (95% CI) †	RR (95%CI)		No. cases, incidence (95% CI) †	RR (95% CI)
Early-onset disease								
Proration of unknown exposure‡								
Unexposed	124, 1.24 (1.03–1.48)	1.69 (1.28–2.24)		103, 1.03 (0.84–1.25)	1.43 (1.03–1.97)		21, 0.21 (0.13–0.32)	3.00 (1.63–5.58)
Exposed	90, 2.10 (1.69–2.58)			63, 1.47 (1.13–1.88)			27, 0.63 (0.41–0.92)	
Unknown, assume exposed								
Unexposed	104, 1.04 (0.85–1.26)	2.47 (1.87–3.26)		ND			ND	
Exposed	110, 2.57 (2.11–3.09)			ND			ND	
Unknown, assume unexposed								
Unexposed	139, 1.39 (1.17–1.64)	1.26 (0.94–1.68)		ND			ND	
Exposed	75, 1.75 (1.38–2.19)			ND			ND	
Late-onset disease								
Proration of unknown exposure‡								
Unexposed	74, 0.74 (0.58–0.93)	3.18 (2.34–4.36)		27, 0.27 (0.18–0.39)	3.37 (2.01–5.73)		47, 0.47 (0.35–0.62)	3.08 (2.07–4.60)
Exposed	101, 2.36 (1.92–2.86)			39, 0.91 (0.65–1.24)			62, 1.45 (1.11–1.85)	
Unknown, assume exposed								
Unexposed	62, 0.62 (0.48–0.79)	4.25 (3.09–5.89)		ND			ND	
Exposed	113, 2.64 (2.17–3.17)			ND			ND	
Unknown, assume unexposed								
Unexposed	89, 0.89 (0.71–1.09)	2.25 (1.66–3.07)		ND			ND	
Exposed	86, 2.01 (1.61–2.48)			ND			ND	
Early-onset plus late-onset disease, exposed vs. unexposed	2.25 (1.84–2.76)			1.83 (1.40–2.39)			3.05 (2.20–4.25)	

*RR, relative risk; ND, not done. †Incidence = no. cases/1,000 live births. ‡Based on prevalence of HIV exposure among those tested.

### Invasive GBS Disease and HIV Exposure

Maternal HIV infection status was available for 327 (84.1%) GBS case-patients, of whom 161 (49.2%) were HIV-exposed (Technical Appendix Table 1). HIV-exposure data were unavailable for 12.3% of EOD and 11.9% of LOD case-patients. Regimens to prevent mother-to-child HIV transmission were documented in only 41.6% of the infants’ medical records and were therefore not analyzed in this study. HIV PCR results were available for 46 (28.6%) of 161 HIV-exposed infants: 6 had EOD (all nonreactive results) and 40 had LOD (8 [20.0%] had reactive results).

Infants with LOD were more likely than those with EOD to be HIV-exposed (58.1% vs. 41.9%; p = 0.004) (Technical Appendix Table 1). HIV-exposed and -unexposed case-patients overall or when stratified by EOD and LOD did not differ substantially with regard to mode of delivery, preterm birth, and low birthweight (Technical Appendix Table 1) or to exposure to meconium-stained liquor, prolonged rupture of membranes, or IAP during labor (data not shown).

The incidence of invasive GBS disease was 2.25-fold (95% CI 1.84–2.76) greater in HIV-exposed than HIV-unexposed infants (4.46 cases/1,000 live births [95% CI 3.85–5.13] vs. 1.98 cases/1,000 live births [95% CI 1.71–2.28]) (Table 1). The higher incidence of GBS disease in HIV-exposed compared with HIV-unexposed infants was evident for EOD case-patients (2.10 vs. 1.24 cases/1,000 live births, respectively; risk ratio 1.69, 95% CI 1.28–2.24) and more so for LOD case-patients (2.36 vs. 0.74 cases/1,000 live births, respectively; risk ratio 3.18, 95% CI 2.34–4.36). Bacteremia and meningitis incidence was also higher in HIV-exposed than HIV-unexposed infants (Table 1). These differences in incidence of invasive GBS disease remained significant in all sensitivity analyses in which missing maternal HIV infection status were extrapolated (Table 1), except for EOD when infants with unknown HIV-exposure status were assumed to be HIV-unexposed (Table 1).

### Factors Associated with Death among Infants with Invasive GBS

The overall case-fatality rate (CFR) was 16.9%; the CFR for meningitis (24.3%) was 2.4-fold greater than that for bacteremia (11.8%; odds ratio 2.24, 95% CI 1.33–4.35; p = 0.0015). The median duration of hospitalization was 1 day for infants who died and 15 days for those who survived (Technical Appendix Table 1). In univariate analysis, meningitis and very low birthweight (<1,500 g) were associated with a higher overall CFR (p = 0.002 and p = 0.003, respectively). Prematurity (<33 weeks’ gestation) was associated with a higher CFR in EOD but not LOD case-patients (p = 0.008 and p = 0.68, respectively) (Technical Appendix Table 2).

### Antimicrobial Drug Susceptibility and Serotyping of GBS

Antimicrobial drug sensitivity profiles were available for 385 (98.9%) of 389 isolates; all were penicillin sensitive. Macrolide resistance was prevalent in 15 (5.5%) of 273 of isolates.

**Figure 2:**
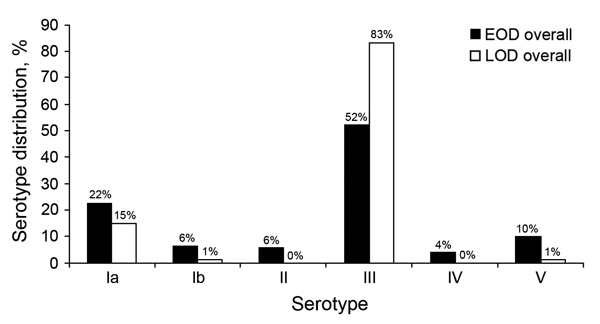
Group B *Streptococcus* serotype distribution among 125 patients with early-onset disease (EOD) and 88 patients with late-onset disease (LOD), Soweto, South Africa, 2004–2008.

Of 389 isolates, 213 (54.8%) were available for serotyping, including 125 (58.6%) from EOD case-patients and 88 (41.3%) from LOD case-patients. The proportion of isolates available for serotyping increased each year: 2004, 15.6%; 2005, 45.1%; 2006, 65.8%; 2007, 63.3%; 2008, 75.0%. Overall, serotypes Ia and III accounted for 84.0% of all serotypes (19.2% [41/213] and 64.8% [138/213], respectively) and for 74.4% of EOD and 97.7% of LOD cases (p<0.001; Figure 2). Serotype distribution remained similar throughout the study and did not differ by HIV-exposure status (data not shown). Overall, a greater proportion of meningitis than bacteremia cases were caused by serotype III (77.8% [63/81] vs. 56.8% [75/132]; p = 0.002), and serotype V was more commonly identified in bacteremia than meningitis cases (7.6% [10/132] vs. 3.7% [3/81]; p = 0.25; Technical Appendix Figure). Serotype distribution between survivors and nonsurvivors did not differ (p = 0.51; data not shown).

### Nationwide Number and Potential Vaccine-Preventable Fraction of Invasive GBS Disease

We estimated ≈3,178 invasive GBS cases and 549 GBS-associated deaths among South Africa’s 2012 birth cohort (Table 2). For HIV-unexposed and -exposed infants, these estimates represent ≈1,639 and 1,544 cases and 283 and 266 deaths, respectively. On the basis of the predefined estimated efficacy of trivalent GBS vaccine to protect infants born at >33 weeks’ gestation, we estimated that, each year, 1,230 cases of invasive GBS disease and 163 deaths could be prevented in HIV-unexposed infants and 886 cases and 117 deaths could be prevented in HIV-exposed infants (Table 2).

**Table 2:** Estimated annual number of invasive GBS disease cases and associated deaths and potential annual vaccine-preventable fraction, South Africa*

National estimates	Overall			HIV unexposed†			HIV exposed†	
	No.	Incidence (95% CI)		No.	Incidence (95% CI)		No.	Incidence (95% CI)
Births	1,168,403‡			823,724			34,4679	
Invasive GBS cases	3,178§	2.72 (2.62–2.81)		1,639¶	1.99 (1.89–2.09)		1,544#	4.48 (4.26–4.71)
Invasive GBS-associated deaths								
No total**	549	0.47 (0.43–0.51)		283	0.34 (0.30–0.39)		266	0.77 (0.68–0.87)
No. in infants born at >33 weeks’ gestation††	420	0.36 (0.33–0.40)		217	0.26 (0.23–0.30)		204	0.59 (0.51–0.68)

Vaccine-preventable cases and deaths								
Trivalent GBS-CV‡‡								
Cases	2,105§§	1.80 (1.73–1.88)		1,230¶¶	1.49 (1.14–1.58)		886##	2.57 (2.40–2.75)
Deaths	278§§	0.24 (0.21–0.27)		163¶¶	0.20 (0.17–2.31)		117##	0.34 (0.28–0.41)
Pentavalent GBS-CV***								
Cases	2,317§§	1.99 (1.90–2.07)		1,354¶¶	1.64 (1.56–1.73)		976##	2.83 (2.66–3.01)
Deaths	306§§	0.26 (0.23–0.29)		179¶¶	0.22 (0.19–0.25)		129##	0.37 (0.31–0.44)

*GBS, group B *Streptococcus;* GBS-CV, GBS polysaccharide–protein conjugate vaccine. †HIV-exposed and -unexposed values were calculated on the basis of national HIV prevalence in pregnant women (29.5%). Incidence values represent cases/1,000 live births. ‡2012 live births. §Overall GBS incidence is 2.72/1,000 live births. ¶GBS incidence for HIV-unexposed infants is 1.99/1,000 live births. #GBS incidence in HIV-exposed infants is 4.48/1,000 live births. **Total deaths in infants <90 days old, assuming 15.2% were born at <33 weeks of gestation and have a case-fatality rate (CFR) of 26.5% and assuming 84.8% were born at >33 weeks of gestation and have a CFR of 15.6%. ††Deaths in infants <90 days old who were born at >33 weeks of gestation (84.8% of infants); CFR 15.6%. ‡‡Trivalent GBS-CV contains serotypes Ia, Ib, and III, which account for 88.3% of cases. §§Assuming vaccine efficacy of 75%. ¶¶Assuming vaccine efficacy of 85%. ##Assuming vaccine efficacy of 65%. ***Pentavalent GBS-CV contains serotypes Ia, Ib, II, III, and V, which account for 97.2% of cases.

## Discussion

We report a high overall incidence of invasive GBS disease in Soweto (2.72 cases/1,000 live births), which is greater than global (0.53 cases/1,000) and African (1.21 cases/1,000) incidence estimates reported in a meta-analysis of studies conducted during 2000–2011 (*3*). That overall estimate included an incidence of 1.98 cases/1,000 live births (95% CI 1.71– 2.28) among HIV-unexposed infants, which is similar to or greater than incidences reported in many resource-rich countries before the widespread use of IAP (*20*). Furthermore, the observed overall incidence was similar to that for the same population a decade earlier (3.0 cases/1,000 live births) (*21*) and to that for women of South Asian descent in South Africa during the 1980s (2.65 cases/1,000 live births) (*22*). Despite the 2007 implementation of a risk-based IAP strategy at CHBAH, the high incidence of GBS disease has persisted, indicating the limited effect of the strategy in a resource-restricted setting with high maternal HIV infection prevalence. The limited effect could indicate poor strategy adherence or that the strategy missed most women whose newborns were at risk for EOD.

The high incidence of invasive GBS disease in South Africa contrasts with the lower incidence reported in South Asia and the Western Pacific, despite similarity in prevalence of maternal vaginal GBS colonization at delivery (*6*). Possible reasons for this discrepancy include differences in delivery location, presence of trained birth-care attendants, and access to health facilities with adequate capability to diagnose and treat invasive GBS disease. Possible reasons for differences are exemplified by the findings in a study conducted in Bangladesh (*23*), which reported that only 1 of 30 culture-confirmed neonatal bacteremia cases was caused by GBS; however, >50.0% of the 259 reported neonatal deaths (many among infants born outside health care facilities) occurred within 24 h of birth, and 62.0% of those cases were not investigated for bacteremia. Our finding that 64.5% of EOD cases were diagnosed within 24 h of birth highlights the effect that births outside of health care facilities or where there is limited capacity for investigating invasive disease in newborns could have on measuring the incidence of EOD. Also, even though case-patients were treated in a secondary–tertiary hospital, death caused by invasive GBS disease was rapid (median 1 d from hospitalization).

In our study, the overall incidence of GBS disease in HIV-exposed infants was 4.46 cases/1,000 live births. The high prevalence of maternal HIV infection is contributing to the high incidence of GBS disease in South Africa, which corroborates data from a Belgium study that reported an incidence of 15.5 cases/1,000 live births (i.e., 5 cases/322 infants, predominantly LOD) in HIV-exposed infants, compared with 0.8 cases/1,000 live births (i.e., 16 cases/20,158 infants) in HIV-unexposed infants (*24*). Our study was not designed to evaluate whether the lower threshold used for investigating for sepsis in HIV-exposed than for HIV-unexposed infants may have contributed to ascertainment bias. However, such bias is unlikely because the threshold for investigating for sepsis among neonates is low in general at CHBAH; an investigation is done only when clinically indicated.

In addition to the higher incidence of LOD observed in HIV-exposed compared with HIV-unexposed infants, we observed that risk for EOD was 1.69-fold (95% CI 1.28–2.24) greater in HIV-exposed infants. This increased risk was present despite our previous observation that the prevalence of vaginal GBS colonization at delivery was lower in HIV-infected than HIV-uninfected women (17.0% vs. 23.0%; p = 0.002), even though rates of vertical colonization were similar for their newborns (52.0%–58.0%) (*25*). Although our study did not identify other differences in prevalence of risk factors for invasive GBS disease between HIV-exposed and -unexposed infants (*26*), we did not have population-level data on prevalence rates of these maternal risk factors for HIV-infected and -uninfected women. However, the observation of a greater difference in risk for LOD than EOD in HIV-exposed newborns compared with HIV-unexposed newborns suggests that risk factors other than peripartum EOD–associated risk factors likely contribute to the heightened susceptibility of invasive disease in HIV-exposed infants. We were unable to determine whether HIV infection in the neonates contributed to an enhanced susceptibility to invasive GBS disease. None of the newborns with EOD for whom HIV testing was done were HIV-positive, whereas 20.0% (8/40) of tested LOD case-patients were HIV-positive. The population-based vertical transmission rate of HIV during the course of the study was 9.6% (*27*). A further limitation of our study was the lack of data on the clinical, immunologic, and HIV-virologic characteristics of the HIV-infected women and analysis of whether these characteristics could have contributed to a heightened susceptibility of invasive disease in their neonates.

Multiple studies have reported an inverse association between maternal GBS serotype–specific antibody levels and an infant’s risk for EOD and LOD (*28*). Lower levels of maternally derived antibodies to several childhood vaccine epitopes have been reported in HIV-exposed but uninfected infants at birth to at least 6 weeks of age (*29*,*30*). Thus, it is plausible that lower naturally acquired capsular antibody in HIV-infected women may contribute to increased susceptibility to invasive GBS disease in HIV-exposed infants; this possibility warrants further investigation. The increased incidence of invasive GBS disease in HIV-exposed but uninfected infants could also be due to observed perturbations of their immune systems (*31*,*32*).

The serotype distribution of GBS isolates from EOD and LOD cases in this study was similar to that observed previously (*21*) and did not differ by HIV-exposure status. Serotypes Ia, Ib, and III, which are included in the current investigational trivalent GBS-CV, covered 78% of EOD and 100% of LOD invasive isolates in this study. However, the overall potential disease reduction of a vaccine against invasive GBS disease may be lower than this potential coverage because the vaccine is unlikely to confer protection through antibody acquisition in neonates born at <34 weeks’ gestation (*33*). The protection of premature newborns against EOD would require prevention of GBS acquisition from the mother. This strategy is pertinent to settings like ours, in which 29.8% of all EOD-associated and 31.4% of all GBS-associated deaths occurred in premature infants despite the rate of premature birth in the community being only 18.0%.

Using a conservative approach of assuming no efficacy against invasive GBS disease in infants born at <33 weeks’ gestation and vaccine efficacy of 85% and 65% in HIV-unexposed and HIV-exposed infants, respectively, we estimate that vaccination of pregnant women with the current investigational trivalent conjugate vaccine could potentially prevent 3,178 GBS cases and 549 GBS-associated deaths annually in South Africa. An effective GBS vaccine could also be used to probe the possible role of GBS in causing sickness and death in countries with limited epidemiologic laboratory capacity (*34*) and in causing stillbirths (*35*).

Technical Appendix Demographics of and outcomes for infants with invasive group B *Streptococcus* (GBS) disease and GBS serotype distribution by clinical presentation and infant factors associated with mortality due to invasive GBS disease.
