# Supplementary material for: Increased Risk for Group B Streptococcus Sepsis in Young Infants Exposed to HIV, Soweto, South Africa, 2004–2008
Source: Emerg Infect Dis. 2015 Apr;21(4):638–45. doi: 10.3201/eid2104.141562 (PMC4378461; doi:10.3201/eid2104.141562)
Supplement: Technical Appendix — Demographics of and outcomes for infants with invasive group B Streptococcus (GBS) disease and GBS serotype distribution by clinical presentation and infant factors associated with mortality due to invasive GBS disease. [file 14-1562-Techapp-s1.pdf]

Article DOI: http://dx.doi.org/10.3201/eid2104.141562

Increased Risk for Group B *Streptococcus* Sepsis in Infants Exposed to HIV, Soweto, South Africa, 2004–2008

Technical Appendix

Technical Appendix Table 1. Demographics and outcomes of young infants with invasive Group B *Streptococcus* disease

Characteristic	No. overall/no. total (%), n = 372 cases	No. with early-onset disease/no. total (%), n = 204*			No. with late-onset disease/no. total, n = 168†		
		Overall	HIV-unexposed	HIV-exposed	Overall	HIV-unexposed	HIV-exposed
Male sex	198/371 (53.4)	109/ 204 (53.4)	50/103 (48.5)	42/ 73 (57.5)	89/167 (53.3)	35/62 (56.5)	45/85 (52.9)
HIV exposed	161/327 (49.2)	75/179 (41.9)			86/148 (58.1)		
Birthweight, g							
Median (range)	2,795 (605–4,300)	2,755 (605–4,240)	2,897.5 (710–4,240)	2,660 (605–4,220)	2,800 (760–4,300)	2,850 (820–4,300)	2,785 (1,270–4,245)
<1,500	37/322 (11.5)	24/ 186 (12.9)	10/ 94 (10.6)	10/ 71 (14.1)	13/136 (9.6)	7/55 (12.7)	5/67 (7.5)
1,500 – <2,500	80/322 (24.8)	48/ 186 (25.8)	22/ 94 (23.4)	20/ 71 (28.2)	32/136 (23.5)	10/55 (18.2)	20/67 (29.9)
≥2,500	205/322 (63.7)	114/ 186 (61.3)	62/94 (66.0)	41/ 71 (57.7)	91/136 (66.9)	38/55 (69.1)	42/67 (62.7)
Gestational age, wk							
≤33	59/320 (18.4)	34/181 (18.8)	19/96 (19.8)	11/68 (16.2)	25/139 (18.0)	10/57 (17.5)	11/68 (16.2)
>33 – <37	26/320 (8.1)	20/181 (11.0)	9/96 (9.4)	8/68 (11.8)	6/139 (4.3)	2/57 (3.5)	4/68 (5.9)
≥37	235/320 (73.4)	127/181 (70.2)	68/96 (70.8)	49/68 (72.1)	108/139 (77.7)	45/57 (78.9)	53/68 (77.9)
Caesarean delivery	64/331 (19.3)	42/190 (22.1)	24/96 (25.0)	13/73 (17.8)	22/141 (15.6)	7/57 (12.3)	12/70 (17.1)
Death							
All infants	63/372 (16.9)	30/ 204 (14.7)	11/ 102 (10.8)	12/ 74 (16.2)	33/168 (19.6)	10/62 (16.1)	18/ 85 (21.2)
Infants with bacteremia	26/220 (11.8)	18/156 (11.5)	7/84 (8.3)	6/50 (12.0)	8/64 (12.5)	0/24 (0)‡	6/34 (17.6)‡
Infants with meningitis	37/152 (24.3)	12/48 (25.0)	4/14 (28.6)	4/24 (16.7)	25/104 (24.0)	10/38 (26.3)	12/51 (23.5)
Median length, d, of hospitalization (range)							
All infants	15.0 (0–216)	13.0 (0–216)	12.0 (1–216)	13.0 (0–67)	20.0 (2–66)	16.5 (2–55)	20.0 (4–66)
Infants who died in hospital	1.0 (0–93)	–0.5 (0–93)	2.0 (0–29)	2.0 (0–93)	1.0 (0–72)	1.0 (0–44)	2.0 (0–72)

*In infants aged <7 d.
†In infants aged 7–90 d.
‡ p value for HIV-exposed vs. HIV unexposed was significant at p = 0.02.

Technical Appendix Table 2. Infant factors associated with mortality due to invasive Group B *Streptococcus* disease*

	Overall				Early-onset disease				Late-onset disease			
Characteristic	No. survived/ no. total (%)	No. died/ no. total (%)	Univariable OR (95% CI)	p value	No. survived/ no. total (%)	No. died/no. total (%)	Univariable OR (95% CI)	p value	No. survived/ no. total (%)	No. died/no. total (%)	Univariable OR (95% CI)	p value
Birth weight, g						N=28				N=24		
<1,500	23/266 (8.7)	13/52 (25.0)	3.26 (1.49–7.13)	0.003	12/155 (7.8)	12/28 (42.9)	8.33 (3.07–22.64)	<0.001	11/111 (9.9)	1/24 (4.2)	0.37 (0.04–3.04)	0.35
1,500–2,499	70/266 (26.3)	9/52 (17.3)	0.74 (0.33–1.64)	0.46	43/155 (27.7)	4/28 (14.3)	0.78 (0.24–2.54)	0.67	27/111 (24.3)	5/24 (20.8)	0.75 (0.25–2.22)	0.61
≥2,500	173/266 (65.0)	30/52 (57.7)	Ref		100/155 (64.5)	12/28 (42.9)	Ref		73/111 (65.8)	18/24 (75.0)	Ref	
Gestational age, wk												
≤33	24/265 (9.1)	2/51 (3.9)	1.79 (0.89–3.61)	0.10	19/154 (12.3)	1/24 (4.2)	3.55 (1.40–9.1)	0.008	5/111 (4.5)	1/27 (3.7)	0.78 (0.24–2.52)	0.68
>33 to <37	44/265 (16.6)	14/51 (27.5)	0.47 (0.11–2.07)	0.47	24/154 (15.6)	10/24 (41.7)	0.45 (0.06–3.64)	0.45	20/111 (18.0)	4/27 (14.8)	0.78 (0.09–7.04)	0.83
≥37	197/265 (74.3)	35/51 (68.6)	Ref		111/154 (72.1)	13/24 (54.2)	Ref		86/111 (77.5)	22/27 (81.5)	Ref	
HIV exposed	129/272 (47.3)	30/51 (58.8)	1.58 (0.86–2.90)	0.14								
Male sex	160/304 (52.6)	37/63 (58.7)	1.28 (0.73–2.22)	0.38								
Early-onset disease (%)	174/309 (56.3)	30/63 (47.6)	Ref									
Late-onset disease (%)	135/309 (43.7)	33/63 (52.4)	1.42 (0.82–2.44)	0.21								
Site of infection												
Bacteremia only	194 (62.8)	26 (41.3)	Ref									
Meningitis	115 (37.2)	37 (58.7)	2.40 (1.38–4.17)	0.002								

*OR, odds ratio; Ref, reference.

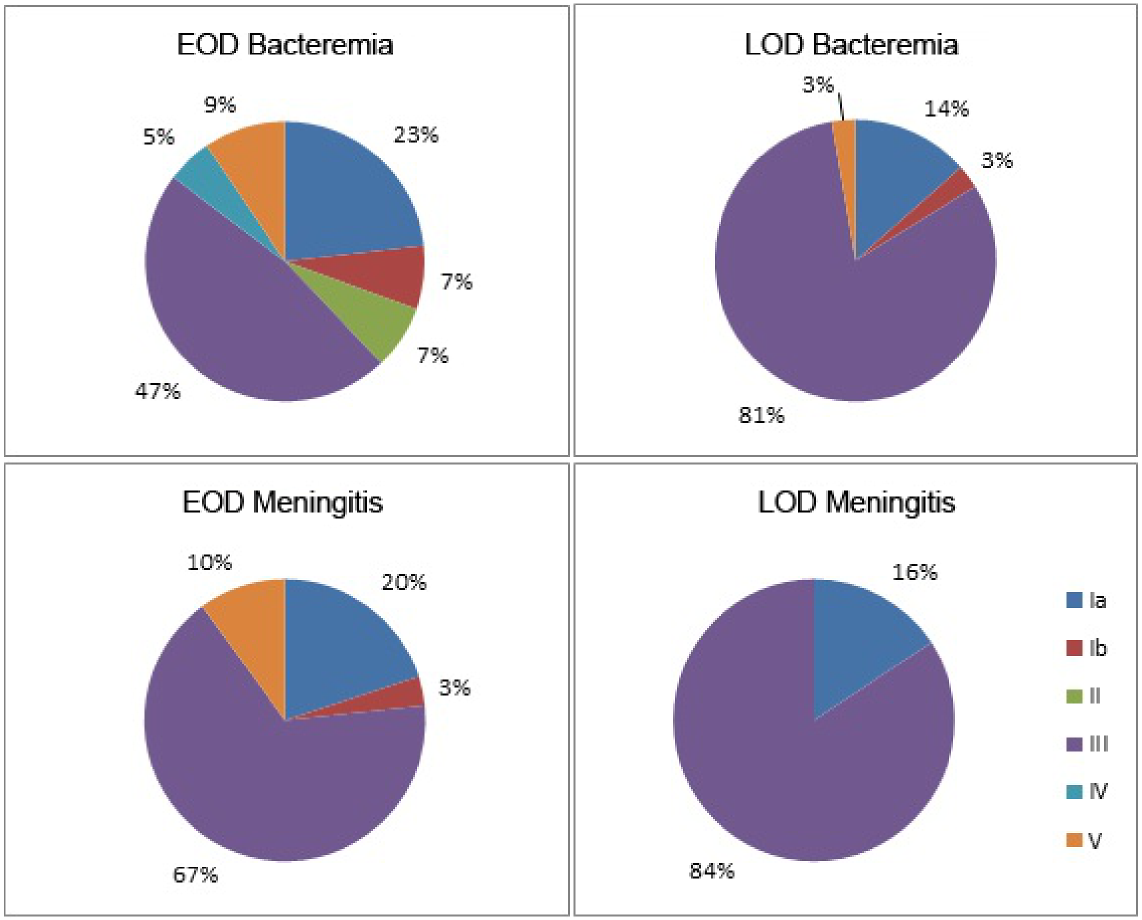

Technical Appendix Figure. Group B *Streptococcus* serotype distribution by clinical presentation. EOD, early-onset disease; LOD, late-onset disease. Serotypes are indicated in the key.
